# Fish Hook as Foreign Body: Not All Foreign Bodies Can Be Fished Out of the Esophagus With Endoscopy Alone

**DOI:** 10.7759/cureus.28164

**Published:** 2022-08-19

**Authors:** Sandra K Rabat, Archana Sridhar, Aamir Makda, Mark M Aloysius

**Affiliations:** 1 Internal Medicine, The Wright Center for Graduate Medical Education, Scranton, USA

**Keywords:** unusual foreign body, foreign body removal, fishhook, fishhook removal, esophageal foreign body

## Abstract

A 75-year-old male presented to the hospital with acute onset of neck pain. Although the patient did not report known ingestion of a foreign body, there was evidence of a fish hook in the cervical esophagus on plain neck radiography. Due to the location at the upper esophageal sphincter in the hypopharynx, the foreign body was not retrievable by endoscopy alone and required better visualization and airway protection with direct laryngoscopy and rigid esophagoscopy. A fish hook was promptly retrieved within 24 hours of the patient's presentation and his symptoms resolved without complications. We report this unusual case to emphasize the importance of proper food preparation, thoroughly chewing food before swallowing, and prompt management of foreign body ingestion in adults.

## Introduction

Esophageal foreign bodies (EFBs) represent a relatively common and urgent clinical condition affecting all ages, which may lead to morbidity and mortality [1,2]. A retrospective study of 310 patients with EFBs demonstrated that the most common foreign objects ingested in adults were meat bolus and fish bone [3]. It is much less common to find a fish hook as an EFB. Sharp foreign bodies (FBs) pose significant risk irrespective of size due to their ability to perforate and cause other complications [4]. We present an unusual case of a 75-year-old patient who presented initially with acute onset of neck pain after consuming a fish. Neck X-ray revealed a fish hook FB in the cervical esophagus, requiring direct laryngoscopy and rigid esophagoscopy for FB removal. This case underlines the importance of proper food preparation and thoroughly chewing food before swallowing. Furthermore, urgent management of FB ingestion in adults is crucial for good outcomes without complications.

## Case presentation

**Figure 1:**
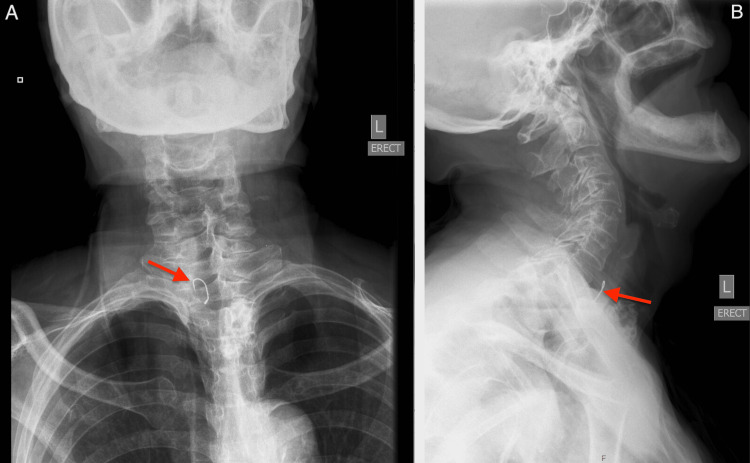
Anterior-posterior (A) and lateral (B) soft tissue neck X-rays demonstrating a bar metallic hook, consistent with a fish hook, present in the soft tissues of the cervical esophagus. The airway is patent and lung apices are clear.

A 75-year-old male with a medical history of cerebral vascular accident three years ago, hypertension, hyperlipidemia, hypothyroidism, and gastroesophageal reflux disease presented to our hospital with neck pain. The patient did not report known ingestion of an FB. He stated that his son caught a fish from the lake with personal fishing equipment. After eating the fish, the patient immediately felt neck pain and presented to the hospital shortly thereafter. He described the pain as non-radiating, rated moderate in intensity, and worsened with swallowing and coughing. On initial presentation, the patient was found to be hemodynamically stable and in no acute distress. Soft tissue neck X-rays revealed a fish hook FB in the cervical esophagus approximately at the level of the cricopharyngeus (Figure 1).

**Figure 2:**
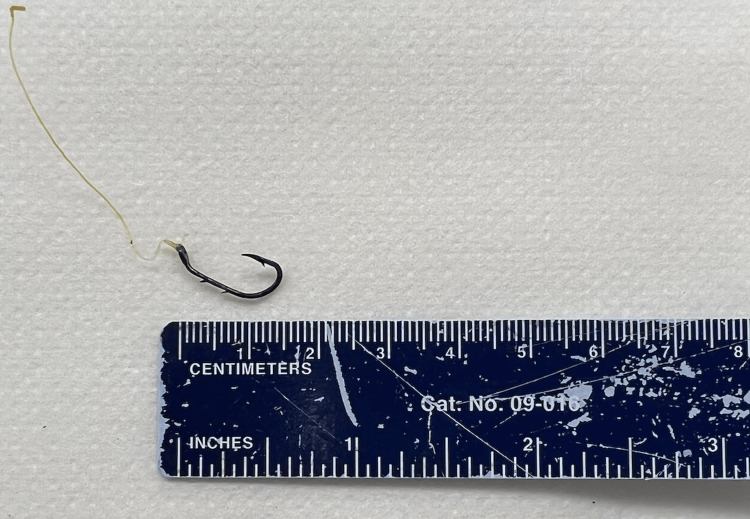
The fish hook after removal.

**Figure 3:**
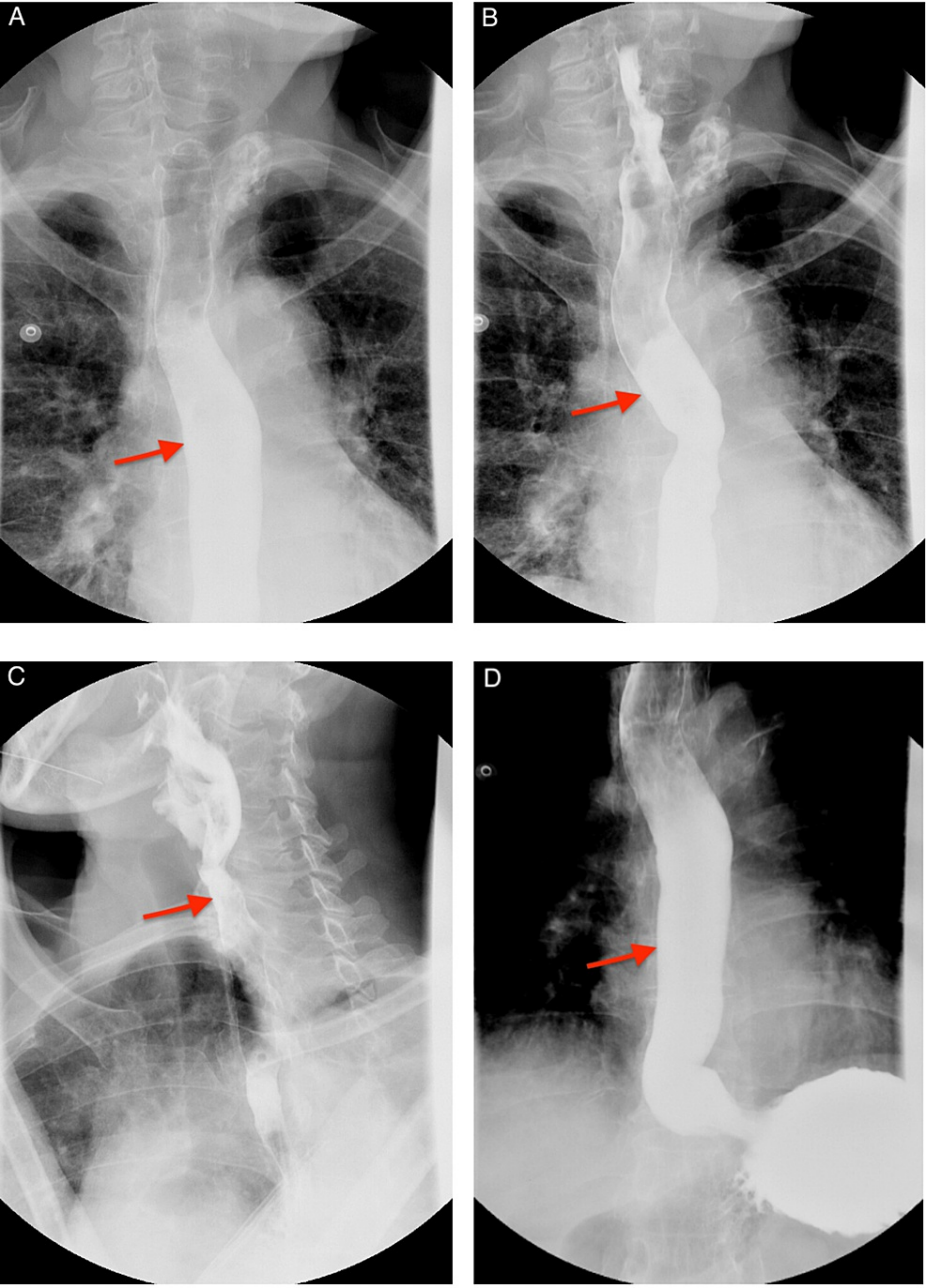
Normal gastrografin swallow study of the esophagus (A-D) after the removal of the fish hook with no esophageal perforation. With the ingestion of gastrografin, a water-soluble contrast, there was no extraluminal leak or mucosal irregularity.

Gastroenterology evaluated the patient, reviewed laboratory and imaging findings, and recommended that the FB object was not amenable to endoscopic intervention with esophagoscopy due to its location at the upper esophageal sphincter in the hypopharynx, requiring better visualization with ENT instruments with airway protection. Otolaryngology was consulted and within 24 hours performed direct laryngoscopy and rigid esophagoscopy with successful removal of FB, resulting in symptom resolution without complication. A gross description of the specimen was provided by pathology confirming it was a steel metal fish hook measuring 1.7 cm long and less than 0.1 cm in diameter with attached white plastic string 4.0 cm long and less than 0.1 cm in diameter with no tissue attached (Figure 2). A gastrografin study was completed thereafter to rule out esophageal perforation, which showed a normal gastrografin swallow examination of the esophagus (Figure 3). The patient had an uneventful recovery and was discharged. 

## Discussion

FB ingestion commonly occurs in children and in high-risk groups of adults, such as those with underlying esophageal disease, acute intoxication, or severe psychiatric disorders [5]. The estimated incidence rate of FB ingestion is 13 per 100,000 individuals and accounts for approximately 1,500 deaths annually in the United States alone [1]. In adults, the esophagus is approximately 20 to 25 cm in length, extending from the hypopharynx to the stomach [6]. FB impaction is most likely to occur in the following sites: upper esophagus followed by the middle esophagus, stomach, pharynx, lower esophagus, and duodenum [2]. Additionally, FBs were more frequently lodged at the narrowest part of the upper esophagus at the cricopharyngeal muscle, especially in patients presenting to the hospital within 24 hours of ingestion [7]. Patients usually present to the hospital with symptoms of odynophagia, dysphagia, FB sensation, chest pain, or nausea and vomiting [6].

The most commonly ingested FBs in children are coins, button batteries, and toys, whereas, in adults, it is usually food boluses, fish bones, or chicken bones [5]. These objects usually result from social activities; in contrast, fish hooks are unusual objects of ingestion [8]. There are very few cases of a fish hook as an EFB reported over the last 20 years [8-11].

**Figure 4:**
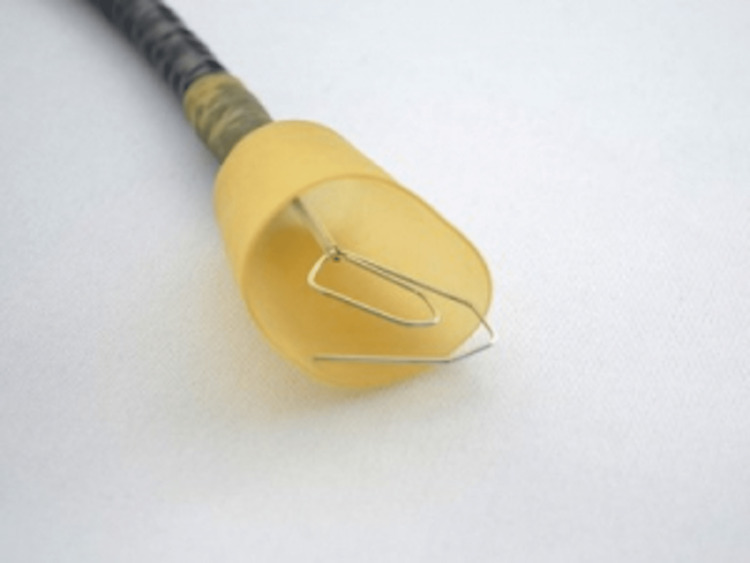
Example of a protective hood for the endoscopic removal of sharp foreign bodies.

**Figure 5:**
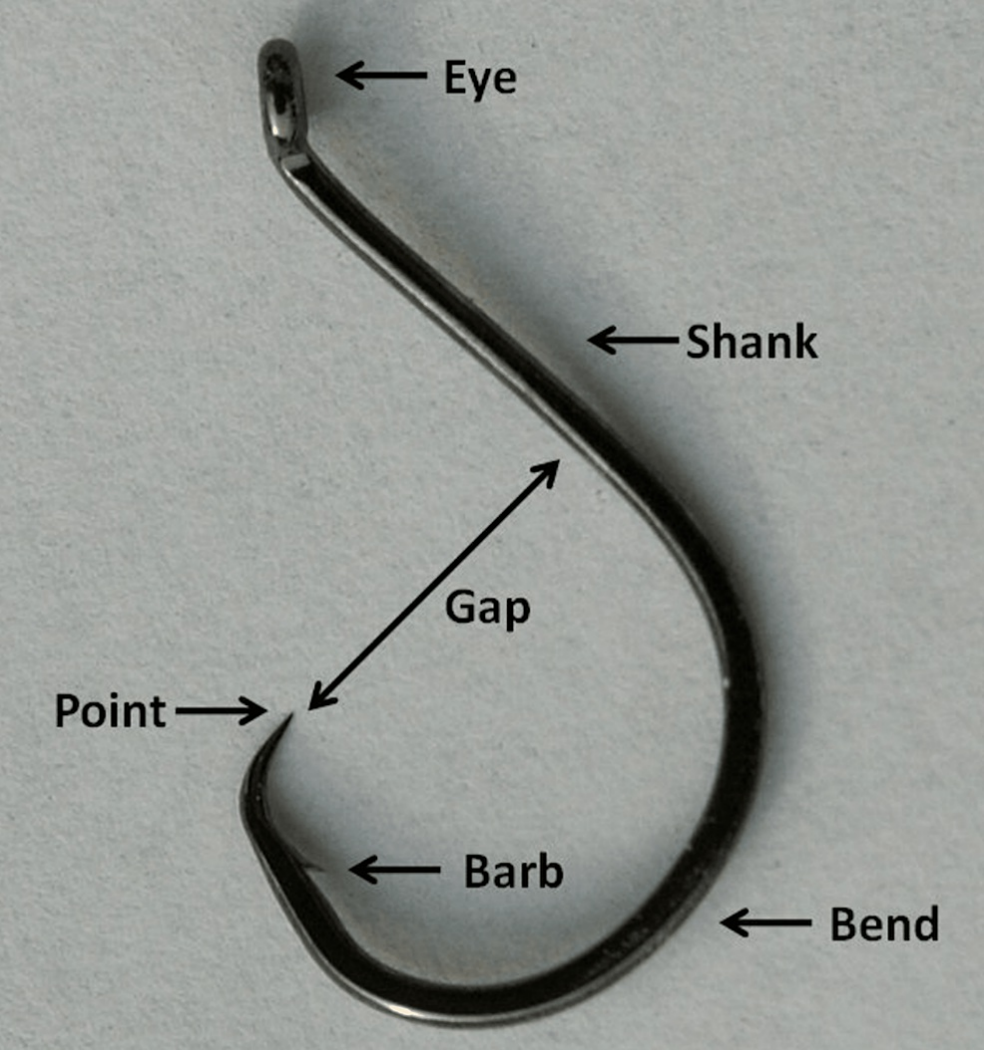
Anatomy of a fish hook.

The majority of ingested FBs pass spontaneously through the gastrointestinal tract, with 10-20% of them requiring endoscopic removal and <1% requiring surgical intervention [12]. Endoscopic management is the first choice in the treatment of EFBs because it is safe, effective, cost-efficient, and avoids the need for surgery [13]. Moreover, a retrospective analysis of 188 inpatient cases with EFBs between 1996-2006 revealed that the majority of cases of FBs were removed via rigid esophagoscopy, with only five cases of FBs removed via surgery [14]. During endoscopic extraction of sharp, pointed FBs, the European Society of Gastrointestinal Endoscopy (ESGE) strongly recommends the use of a protective device to avoid esophagogastric/pharyngeal damage and aspiration [12]. The use of an overtube or a retractable latex-rubber condom-type hood is effective to protect the upper aerodigestive structures and facilitate endoscopy (Figure 4) [15]. Sharp, pointed, and elongated FBs, such as fish bones, are associated with a greater risk of perforation, vascular penetration, or other complications as they are more likely to be embedded in the esophagus [4]. Moreover, this risk likely increases with a fish hook as a FB object due to the anatomy of a fish hook with its point and barb(s), which may pose a challenge to remove (Figure 5). The backward-projecting barb near the hook’s point typically prevents withdrawal of the hook, designed to lodge deep in the flesh of a fish and not intended to be ingested by humans [11]. In our patient’s case, the fish hook retrieved had a sharp point and multiple barbs.

Recent advances aimed at early diagnosis and removal of FBs can improve the survival rates in these patients. Prompt treatment can result in good outcomes, as seen with our patient’s case. However, there are serious potential complications of FB ingestion if treatment is delayed including mediastinitis, paraesophageal abscess, pneumomediastinum, subcutaneous emphysema, pneumothorax, tracheoesophageal fistula, aortoesophageal fistula, aspiration, and asphyxia [4]. Complications and hospital length of stay can be reduced if treatment is initiated within 24 hours of FB ingestion [7]. Some limitations to prompt FB treatment may include social determinants of health such as access to care and transportation to a hospital in underserved communities. A retrospective study revealed that a longer duration for treatment, age greater than 60, and impaction in the esophagus are some of the risk factors for developing complications after FB ingestion [13].

## Conclusions

FB ingestion is a common clinical problem in the United States, with meat boluses and chicken bones identified as the most common FB objects encountered. Fish hook as a FB is unusual and warrants extra precautions in the management due to the increased risk for complications. Although endoscopic procedures have achieved a high success rate and efficacy in the management of EFBs, it’s important to note that the use of a protective device in the removal of sharp FBs is recommended to avoid esophagogastric/pharyngeal damage and aspiration. Furthermore, food must be prepared appropriately, and consumers should observe what they intend to swallow. Patients with FB ingestion should present to the hospital urgently for appropriate management. Complications can be reduced if the treatment is conducted within 24 hours of FB ingestion.

## References

[1] Wu L, Lei G, Liu Y, Wei Z, Yin Y, Li Y, Wang G (2021). Retrospective analysis of esophageal foreign body ingestion: differences among weekday, weekends, and holidays. Risk Manag Healthc Policy.

[2] Yao CC, Wu IT, Lu LS (2015). Endoscopic management of foreign bodies in the upper gastrointestinal tract of adults. Biomed Res Int.

[3] Sittitrai P, Pattarasakulchai T, Tapatiwong H (2000). Esophageal foreign bodies. J Med Assoc Thai.

[4] Boo SJ, Kim HU (2018). [Esophageal foreign body: treatment and complications]. Korean J Gastroenterol.

[5] Bekkerman M, Sachdev AH, Andrade J, Twersky Y, Iqbal S (2016). Endoscopic management of foreign bodies in the gastrointestinal tract: a review of the literature. Gastroenterol Res Pract.

[6] Schaefer TJ, Trocinski D (2022). Esophageal foreign body. https://www.ncbi.nlm.nih.gov/books/NBK482131/.

[7] Zhang X, Jiang Y, Fu T, Zhang X, Li N, Tu C (2017). Esophageal foreign bodies in adults with different durations of time from ingestion to effective treatment. J Int Med Res.

[8] Opoku-Buabeng J, Abdulai R (2012). Unsual foreign body in the throat: a report on 3 cases. J West Afr Coll Surg.

[9] Ogah SA, Olatoke F, Okomanyi A, Igbobu B (2014). Fish hook and line impaction in the esophagus: an unusual and interesting foreign body. IOSR J Dent Med Sci.

[10] Okhakhu AL, Ogisi FO (2007). An unusual foreign body in human oesophagus - case report. Benin J Postgrad Med.

[11] Iwamuro M, Okada H, Kawai D (2009). Endoscopic removal of a fishhook in the esophagus. Gastrointest Endosc.

[12] Birk M, Bauerfeind P, Deprez PH (2016). Removal of foreign bodies in the upper gastrointestinal tract in adults: European Society of Gastrointestinal Endoscopy (ESGE) clinical guideline. Endoscopy.

[13] Wang X, Su S, Chen Y (2021). The removal of foreign body ingestion in the upper gastrointestinal tract: a retrospective study of 1,182 adult cases. Ann Transl Med.

[14] Türkyilmaz A, Aydin Y, Yilmaz O, Aslan S, Eroğlu A, Karaoğlanoğlu N (2009). [Esophageal foreign bodies: analysis of 188 cases]. Ulus Travma Acil Cerrahi Derg.

[15] Sugawa C, Ono H, Taleb M, Lucas CE (2014). Endoscopic management of foreign bodies in the upper gastrointestinal tract: a review. World J Gastrointest Endosc.

